# Metabolomic and transcriptomic studies of improvements in myocardial infarction due to Pycr1 deletion

**DOI:** 10.1111/jcmm.17637

**Published:** 2022-12-10

**Authors:** Zhimin Xue, Yiwen Pan, Xugang Kong, Jiefang Zhang, Danyu Wu, Binquan Zhou

**Affiliations:** ^1^ Department of Cardiology, Sir Run Run Shaw Hospital Zhejiang University School of Medicine Hangzhou China

**Keywords:** coronary heart disease, metabolomics, myocardial infarction, proline metabolism, pyrroline‐5‐carboxylate reductase 1

## Abstract

Myocardial infarction (MI) remains a major challenge to cardiovascular health worldwide, with poor healing leaving a direct impact on patients' quality of life and survival. Metabolic abnormalities after MI are receiving increasing attention. Our previous studies showed that enhancing proline catabolism ameliorates hypoxic damage to myocardial cells; therefore, we sought to determine whether reducing the synthesis of endogenous proline also affects MI. We analysed GEO datasets associated with MI and western blot of mouse heart tissue in an MI model to demonstrate pyrroline‐5‐carboxylate reductase 1 (Pycr1) expression level after MI. We constructed Pycr1 KO mice by CRISPR/Cas9 technology to explore the effect of Pycr1 gene KO after MI using transcriptomic and metabolomic techniques. In this study, we found reduced mRNA and protein expression levels of Pycr1 in the hearts of mice after MI. We observed that Pycr1 gene KO has a protective effect against MI, reducing the area of MI and improving heart function. Using transcriptomics approaches, we found 215 upregulated genes and 247 downregulated genes after KO of the Pycr1 gene, indicating that unsaturated fatty acid metabolism was affected at the transcriptional level. Metabolomics results revealed elevated content for 141 metabolites and decreased content for 90 metabolites, among which the levels of fatty acids, glycerol phospholipids, bile acids, and other metabolites increased significantly. The changes in these metabolites may be related to the protective effect of Pycr1 KO on the heart after MI. Pycr1 gene KO has a protective effect against MI and our research will lay a solid foundation for the development of future Pycr1‐related drug targets.

## 1 INTRODUCTION

Myocardial infarction (MI) is the most dangerous cardiovascular disease worldwide today.^1^ With the continuous improvement of several treatment methods, such as percutaneous coronary intervention, the number of immediate deaths caused by acute MI has decreased significantly. However, there is an increased rate of hospitalizations and deaths due to post‐MI ventricular remodelling; moreover, the clinical manifestation of the continuous change in size, morphology, structure, and function of the post‐MI ventricles is the main factor determining the incidence of post‐AMI cardiac events and long‐term prognosis, and there is still a lack of effective countermeasures.^2^ Therefore, elucidating the characteristics and mechanisms of post‐MI ventricular remodelling and exploring effective targeted therapeutic targets are important issues facing cardiovascular disease researchers. It has been established that the heart undergoes a series of metabolic changes called “metabolic remodelling” in response to AMI‐induced myocardial ischemia, and the onset and progression of ventricular remodelling are closely related to the occurrence of cardiac metabolic disorders such as glucosolipid metabolism disorders.^3^ However, the relationship between amino acid metabolism and post‐AMI ventricular remodelling has rarely been reported. We have confirmed that post‐MI proline metabolism disorders are closely related to ventricular remodelling,^4^ with elevated proline content in the heart after MI and significantly decreased proline catalytic enzyme PRODH expression.^5^ Therefore, our previous studies have shown the stagnation of proline catabolism in the heart after AMI and led us to speculate that reducing endogenous proline synthesis may be beneficial for alleviating post‐AMI ventricular remodelling. However, whether it is possible to alleviate ventricular remodelling by reducing the synthesis of endogenous proline after AMI has not been reported.

P5cr is an important housekeeping protein that is widely expressed in organisms in nature, and its main function is to catalyse the conversion of pyrroline‐5‐carboxylic acid (P5C) into proline, which plays a key role in amino acid metabolism.^6^ P5cr has three isoenzymes: Pycr1, Pycr2, and Pycrl. Pycr1 and Pycr2 are localized to the mitochondria and preferentially associate with NADH as a cofactor, while Pycrl is localized in the cytoplasm and has a higher affinity for NADPH. The Pycr1 protein is a mitochondrial in‐body membrane structural protein that is not only involved in proline metabolism but also closely related to energy metabolism and intracellular oxidative stress levels in the mitochondria.^7^, ^8^ To date, studies have found that Pycr1 is highly expressed in tumour tissues and that its expression is related to the occurrence and development of a variety of tumours, such as prostate cancer, lymphoma, and breast cancer, which has become a potential therapeutic target,^9^, ^10^, ^11^ but the role of Pycr1 in cardiovascular diseases, especially MI, has not been clear.

In this study, we use the GEO public database to analyse the change in Pycr1 expression levels in the heart after MI, use the CRISPR–Cas9‐mediated gene editing method to construct PYCR1 gene knockout (KO) mice, build an MI model in mice by ligating the left anterior descending (LAD) coronary artery to study the effect of Pycr1 KO on postembryonic MI, and finally use state‐of‐the‐art transcriptomics and metabolomics techniques to study the changes in cardiac metabolites and transcriptional levels after Pycr1 KO in these mice. We believe that our findings will lay the groundwork for further exploration of the role of Pycr1 in MI and the future development of new treatment options for this disease.

## 2 MATERIALS AND METHODS

### 2.1 Interbreeding and identification of Pycr1 KO mice

Pycr1 KO mice were obtained from Cyagen Biosciences (Santa Clara, CA, USA). The Pycr1 KO mouse model (C57BL/6N) was created by CRISPR/Cas‐mediated genome engineering. The Pycr1 gene (NCBI Reference Sequence: NM_144795; Ensembl: ENSMUSG00000025140) is located on mouse chromosome 11. Eight exons were identified, with the ATG start codon in exon 1 and the TGA stop codon in exon 7. Exons 3–6 were selected as target sites. Cas9 and gRNA will be coinjected into fertilized eggs for KO mouse production. The pups were genotyped by PCR followed by sequencing analysis. The deletion of the target gene was identified by PCR analysis of genomic DNA isolated from the tails of founder mice. The CRISPR/Cas9‐mediated Pycr1 mutant genes in the C57BL/6 background strains were amplified using primer sets. The Pycr1 gene KO mice were identified by using the sense primer 5′‐GTTCTGGCTGCACACAAGATAATG‐3′ and antisense primer 5′‐CATGTTTCACTCACTCTCACCAG‐3′. The mice that were WT for the Pycr1 gene were identified by the sense primer 5′‐GAGAGGCTAGAACTAGCTTTGAG‐3′ and antisense primer 5′‐CATCACAGACTTCTAAACCACAG‐3′. Thereafter, 10 pmoles of sense and antisense primers were added, and the reaction mixture was subjected to 35 cycles of amplification on a Perkin‐Elmer Thermal Cycler as follows: 30 s, 94°C; 30 s, 60°C; 35 s, 72°C. After amplification, the final PCR products of 402 bp and 556 bp in the tail genomic DNA of KO and WT mice were electrophoresed on 2% agarose gels. Note: Exon 3 starts approximately 14.99% into the coding region. Exons 3–6 cover 71.09% of the coding region. The size of the effective KO region was ~1153 bp. The KO region does not contain any other known gene.

### 2.2 Establishment of the mouse MI model

All animal experiments in this study were conducted with approval from the Animal Care and Use Committee of the Zhejiang University School of Medicine according to the Guide for the Care and Use of Laboratory Animals published by the United States National Institutes of Health (NIH publication No. 85–23, revised 1996). C57BL/6 mice weighing 20–25 g were kept individually at a temperature of 24 ± 2°C under a 12‐h light/12‐h dark cycle with free access to food and water throughout the experiment.

MI was generated in 8‐week‐old mice via surgical ligation of the LAD coronary artery as previously described. Briefly, mice were anaesthetised with 2% isoflurane inhalation. A skin incision was made over the left thorax, and the pectoral muscles were retracted to expose the ribs. The heart was removed through the fourth intercostal space, and the LAD artery was ligated with a 6–0 suture, disrupting flow to the apical myocardium. The heart was immediately placed back into the intrathoracic space after a knot was tied in the suture, followed by manual evacuation of the pneumothorax and closure of the muscle and skin suture. Sham‐operated animals were subjected to the same surgical procedures, except that the suture was passed under the LAD artery but not tied.

### 2.3 GEO dataset analysis

The RNA‐seq dataset GSE114695 contains the mRNA expression levels from mouse left ventricles at 1 day (1 D), 1 week (1 W), and 8 weeks (8 W) after MI induction by permanent ligation of the LAD coronary artery (https://www.ncbi.nlm.nih.gov/geo/query/acc.cgi?acc=GSE114695). The RNA‐seq dataset GSE153485 contains mRNA expression levels in mouse heart tissue samples 1 day after MI or sham operation (https://www.ncbi.nlm.nih.gov/geo/query/acc.cgi?acc=GSE153485). The RNA‐seq dataset GSE153494 contains mRNA expression levels from mouse hearts at different time points (1 day and 3 days) after MI (https://www.ncbi.nlm.nih.gov/geo/query/acc.cgi?acc=GSE153494). The RNA‐seq dataset GSE158415 contains mRNA expression levels from the hearts of mice at 1 day post‐MI (https://www.ncbi.nlm.nih.gov/geo/query/acc.cgi?acc=GSE158415). We compared the mRNA expression levels of the proline synthetase Pycr1 according to the FPKM or RPKM values in the above GEO datasets.

### 2.4 Histological analysis

As described previously, at the end of the experiments, hearts were removed quickly and fixed with 4% formaldehyde for paraffin embedding and sectioning. The hearts were sectioned transversely along the short axis from apex to base into five sections. Masson's trichrome staining was performed at the histology core of UT Southwestern following standard protocols. Slices were prestained with Bouin's solution for 45 min at 55°C and then incubated in Weigert's iron haematoxylin, Biebrich scarlet‐acid fucosine, phosphotungstic/phosphomolybdic acid solution, or aniline blue solution. Then, the tissue samples were differentiated with acetic acid for 2 min, dehydrated with 95% ethanol and absolute ethanol, and finally cleared with xylene and sealed. Images were collected using a bright field microscope, and the infarct area was quantified with ImageJ on Masson's trichome‐stained sections.

### 2.5 Echocardiography

As we described previously, transthoracic echocardiography was performed with a Vevo 1100 system (FUJIFILM VisualSonics, Inc.) equipped with a 30 MHz imaging transducer. Mice were anaesthetised with 2% isoflurane gas for echocardiography after MI and before sacrifice. The body temperature of each mouse was maintained at 37°C with a heating pad, and the heart rate was maintained at approximately 500 beats per min. The left ventricular (LV) functions of the mice, including LV ejection fraction (LVEF), LV fractional shortening (LVFS), LV end‐systolic diameter (LVESD), and LV end‐diastolic diameter (LVEDD), were calculated from M‐mode recordings with Vevo LAB.

### 2.6 Western blot analysis

As we described previously, heart tissue was first lysed in RIPA lysis buffer for 15 min, followed by centrifugation at 4°C for 15 min, and the protein concentration was measured. Then, samples containing the same amount of protein were electrophoresed, transferred to a PVDF membrane after denaturation at 100°C, blocked with 5% skim milk, and incubated overnight at 4°C with β‐actin (#23660‐1‐AP, Proteintech) and Pycr1 (#13108‐1‐AP, Proteintech) primary antibodies. On the following day, the membranes were incubated with HRP‐labelled goat anti‐rabbit IgGs (#7074, CST) at 37°C for 2 h. Finally, the membranes were visualized on a ChemiDoc MP imaging system (Bio–Rad) with enhanced chemiluminescence reagent (Fdbio Science).

### 2.7 Metabolomic analysis

#### 2.7.1 Sample preparation and extraction

Tissue sample class I: The sample was removed from the −80°C refrigerator and thawed on ice. The sample was cut and 20 mg of sample was weighed, homogenized (30 HZ) for 20 s with a steel ball and then centrifuged (1007 *g*, 4°C) for 30 s. Then, 400 μl of 70% methanol in water was added as an internal standard extractant, the sample was shaken (252 *g*) for 5 min and then placed on ice for 15 min. The extracted sample was centrifuged (16,114 *g*, 4°C) for 10 min, and 300 μl of the supernatant was transferred to a separate tube and allowed to stand at −20°C for 30 min. Finally, the sample was centrifuged (16114 *g*, 4°C) for 3 min, and the supernatant was collected for analysis.

T3 UPLC conditions: The sample extracts were analysed using an LC–ESI–MS/MS system (UPLC, ExionLC AD, https://sciex.com.cn/; MS, QTRAP® System, https://sciex.com/). The analytical conditions were as follows: UPLC: column, Waters ACQUITY UPLC HSS T3 C18 (1.8 μm, 2.1 mm*100 mm); column temperature, 40°C; flow rate, 0.4 ml/min; injection volume, 2 μl; solvent system, water (0.1% formic acid):acetonitrile (0.1% formic acid); gradient program, 95:5 V/V at 0 min, 10:90 V/V at 11.0 min, 10:90 V/V at 12.0 min, 95:5 V/V at 12.1 min, 95:5 V/V at 14.0 min. Amide UPLC conditions: The sample extracts were analysed using an LC–ESI–MS/MS system (UPLC, ExionLC AD, https://sciex.com.cn/; MS, QTRAP® System, https://sciex.com/). The analytical conditions were as follows: UPLC: column, Waters ACQUITY UPLC BEH Amide 1.7 μm, 2.1 mm*100 mm; column temperature, 40°C; flow rate, 0.4 ml/min; injection volume, 2 μl; solvent system, water (25 mM ammonium formate/0.4% ammonia):acetonitrile; gradient program, 10:90 V/V at 0 min, 40:60 V/V at 9.0 min, 60:40 V/V at 10.0 min, 60:40 V/V at 11.0 min, 10:90 V/V at 11.1 min, 10:90 V/V at 15.0 min.

ESI‐QTRAP‐MS/MS: T3 and HILIC used the same mass spectrometry parameters. LIT and triple quadrupole (QQQ) scans were acquired on a triple quadrupole‐linear ion trap mass spectrometer (QTRAP), QTRAP® LC–MS/MS system equipped with an ESI Turbo Ion‐Spray interface, operating in positive and negative ion mode and controlled by Analyst 1.6.3 software (Sciex). The ESI source operation parameters were as follows: source temperature 500°C; ion spray voltage (IS) 5500 V (positive), −4500 V (negative); ion source gas I (GSI), gas II (GSII), and curtain gas (CUR) were set at 55, 60, and 25.0 psi, respectively; and the collision gas (CAD) was high. Instrument tuning and mass calibration were performed with 10 and 100 μmol/L polypropylene glycol solutions in QQQ and LIT modes, respectively. A specific set of MRM transitions was monitored for each period according to the metabolites eluted within this period.

#### 2.7.2 Analytical methods

PCA: Unsupervised principal component analysis (PCA) was performed by the statistics function prcomp within R (www.r‐project.org). The data were unit variance scaled before unsupervised PCA. Hierarchical cluster analysis and Pearson correlation coefficients: The hierarchical cluster analysis (HCA) results of samples and metabolites were presented as heatmaps with dendrograms, while Pearson correlation coefficients (PCCs) between samples were calculated by the cor function in R and presented only as heatmaps. Both HCA and PCC were carried out by the R package ComplexHeatmap. For HCA, normalized signal intensities of metabolites (unit variance scaling) are visualized as a colour spectrum. Differential metabolites selected: Significantly differentially abundant metabolites between groups were determined by VIP ≥ 1 and absolute log_2_FC (fold change) ≥ 0.5. VIP values were extracted from the OPLSDA results, which also contained score plots and permutation plots and were generated using the R package MetaboAnalystR. The data were log‐transformed (log2) and mean centered before OPLS–DA. To avoid overfitting, a permutation test (200 permutations) was performed. The KEGG annotation and enrichment analysis: Identified metabolites were annotated using the KEGG Compound Database (http://www.kegg.jp/kegg/compound/), and annotated metabolites were then mapped to the KEGG Pathway Database (http://www.kegg.jp/kegg/pathway.html). Significantly enriched pathways are identified based on the hypergeometric test *p*‐value for a given list of metabolites.

### 2.8 Transcriptomics analysis

#### 2.8.1 Experimental procedures

RNA quantification and qualification RNA integrity was assessed using the Fragment Analyser 5400 (Agilent Technologies). Library preparation for transcriptome sequencing: Total RNA was used as input material for the RNA sample preparations. Sequencing libraries were generated using the NEBNext®UltraT M RNA Library Prep Kit for Illumina (NEB) following the manufacturer's recommendations, and index codes were added to attribute sequences to each sample. Briefly, mRNA was purified from total RNA using poly(T) oligo‐attached magnetic beads. Fragmentation was carried out using divalent cations under elevated temperature in NEBNext First‐Strand Synthesis Reaction Buffer (5X). First‐strand cDNA was synthesized using random hexamer primers and M‐MuLV Reverse Transcriptase (RNase H). Second‐strand cDNA synthesis was then performed using DNA Polymerase I and RNase H. Remaining overhangs were converted into blunt ends via exonuclease/polymerase activities. After adenylation of the 3′ ends of DNA fragments, NEB Next Adaptors with hairpin loop structures were ligated to prepare for hybridization. To preferentially select cDNA fragments 250 ~ 300 bp in length, the library fragments were purified with an AMPure XP System (Beckman Coulter, Beverly, USA). Then, 3 μl of USER Enzyme (NEB, USA) was mixed with size‐selected, adaptor‐ligated cDNA at 37°C for 15 min followed by 5 min at 95°C before PCR. Then, PCR was performed with Phusion High‐Fidelity DNA polymerase, Universal PCR primers and Index (X) Primer. Finally, PCR products were purified (AMPure XP system), and library quality was assessed on the Agilent Bioanalyzer 2100 system. Clustering and sequencing (Novogene Experimental Department): The clustering of the index‐coded samples was performed on a cBot Cluster Generation System using TruSeq PE Cluster Kit v3‐cBot‐HS (Illumina) according to the manufacturer's instructions. After cluster generation, the library preparations were sequenced on an Illumina Novaseq 6000 platform, and 150 bp paired‐end reads were generated.

#### 2.8.2 Bioinformatics analysis

*Data Quality Control—Raw data*: The original fluorescence image files obtained from the Illumina platform were transformed to short reads (raw data) by base calling, and these short reads were recorded in FASTQ format, which contains both sequence information and corresponding sequencing quality information. Evaluation of data (data quality control): Sequence artefacts, including reads containing adapter contamination, low‐quality nucleotides and unrecognizable nucleotides (N), serve as a barrier to subsequent reliable bioinformatics analysis. Hence, quality control is an essential step and is applied to guarantee meaningful downstream analysis. We used Fastp (version 0.19.7) to perform basic statistical analysis of the quality of the raw reads. The steps of data processing were as follows: (1) Discard a paired read if either read contains adapter contamination; (2) discard a paired read if more than 10% of bases are uncertain in either read; and (3) discard a paired read if the proportion of low‐quality (Phred quality < 5) bases is over 50% in either read.

To explore the profiles of DEGs between WT and KO, the identification of statistically significant DEGs and their respective fold‐changes in gene expression level were implemented by an R package. After this, multiple hypothesis testing was performed by first correcting the *p*‐value of each gene to control the false discovery rate (FDR) using the Benjamini–Hochberg procedure. The criteria used for identifying DEGs were a |log_2_fold change| ≥ 0.5 and an FDR < 0.05. To gain insight into the change in phenotype, Gene Ontology (GO) (http://www.geneontology.org/) and Kyoto Encyclopedia of Genes and Genomes (KEGG) (https://www.kegg.jp/) enrichment analyses of annotated differentially expressed genes were performed based on hypergeometric tests. The significance levels of terms and pathways were Bonferroni‐corrected with a rigorous threshold for the *q*‐value (FDR *q*‐value ≤ 0.05).

### 2.9 RNA isolation and real‐time PCR

According to the instruction manual, total RNA was first extracted from the tissue using TRIzol, and total RNA was then identified and quantified with the NanoDrop and Agilent 2100 bioanalyzers and converted to complementary DNA using the RT reagent Kit for real‐time PCR using the PCR mixture and a Halo 480II System (Roche). The mRNA levels were normalized to the β‐actin levels and are reported as fold changes compared with the control group. The sequences of the primers were as follows: Pycr1 (forward, 5′‐GAAGATGGCAGGCTTGTGGA‐3′, reverse, 5′‐CTGGGAAGCCCCATTTTCAC3′) and β‐actin (forward, 5′‐AGGGAAATCGTGCGTGACAT‐3′, reverse, 5′‐CGCAGCTCAGTAACAGTCCG‐3′).

### 2.10 Statistical analysis

Three biological repeats were conducted for all experiments. All the results are expressed as the mean ± SD. Statistical analysis was performed one‐way anova or Student's *t*‐test using GraphPad Prism 8. Differences with a *p*‐value of <0.05 were recognized as statistically significant.

## 3 RESULTS

### 3.1 Pycr1 expression is downregulated in the heart after MI

**FIGURE 1:**
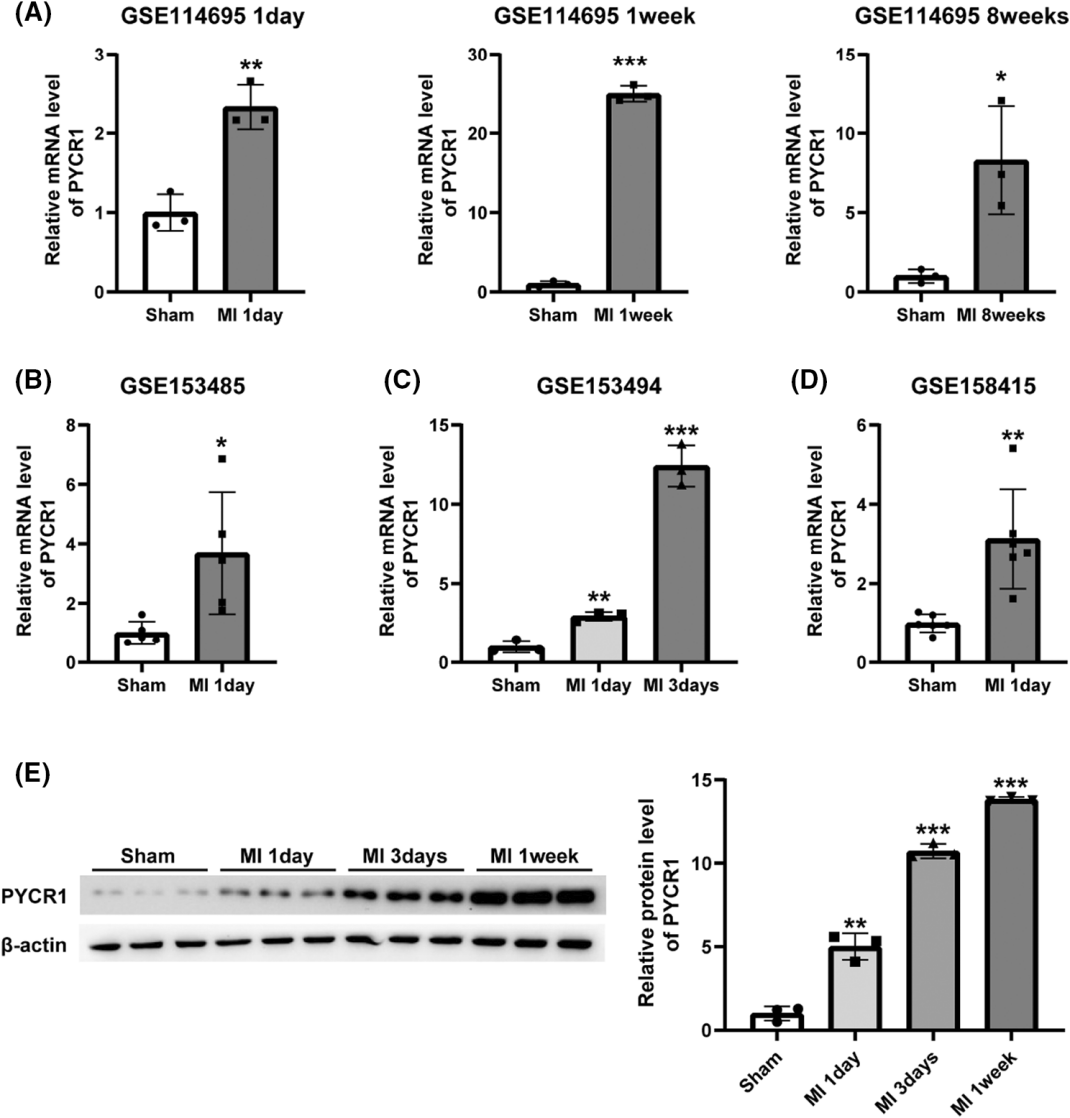
Pycr1 expression is downregulated in the heart after MI. (A) Relative expression levels of the proline synthetase Pycr1 in GSE114695 (*n* = 3). (B) Relative expression levels of the proline synthetase Pycr1 in GSE153485 (*n* = 5). (C) Relative expression levels of the proline synthetase Pycr1 in GSE153494 (*n* = 3). (D) Relative expression levels of the proline synthetase Pycr1 in GSE158415 (*n* = 6). (E) Western blot analysis and quantification of Pycr1 expression at 1, 3 days, and 1 week after MI (*n* = 3). The data are presented as the mean ± SD values. *p < 0.05, **p < 0.01, ***p < 0.001 versus the control group.

The RNA‐seq dataset GSE114695 contains mRNA expression levels in the mouse left ventricles at 1 day (1D), 1 week (1 W), and 8 weeks (8 W) after the induction of MI by permanent ligation of the LAD coronary artery (https://www.ncbi.nlm.nih.gov/geo/query/acc.cgi?acc=GSE114695; Figure 1A). The RNA‐seq dataset GSE153485 contains the mRNA expression levels in mouse heart tissue samples 1 day after MI or sham operation (https://www.ncbi.nlm.nih.gov/geo/query/acc.cgi?acc=GSE153485; Figure 1B). The RNA‐seq dataset GSE153494 contains the mRNA expression levels in mouse hearts at different time points (1 and 3 days) after MI (https://www.ncbi.nlm.nih.gov/geo/query/acc.cgi?acc=GSE153494; Figure 1C). The RNA‐seq dataset GSE158415 contains the mRNA expression levels in the hearts of mice at 1 day post‐MI (https://www.ncbi.nlm.nih.gov/geo/query/acc.cgi?acc=GSE158415; Figure 1D). We compared the mRNA expression levels of the proline synthetase Pycr1 (FPKM or RPKM values) in the above GEO datasets and found that as the time after MI injury increased, Pycr1 expression levels increased, becoming further elevated at 1 day, 3 days, 1 week, and 8 weeks of MI. To verify the change in PYCR1 protein levels after MI, we measured Pycr1 expression levels in the heart at 1 day, 3 days, and 1 week after MI with western blotting and found that their expression levels were also significantly increased, in line with the findings from the GEO public database (Figure 1E).

### 3.2 CRISPR/Cas9‐mediated KO of Pycr1 reduced infarct area and improved cardiac function after MI

**FIGURE 2:**
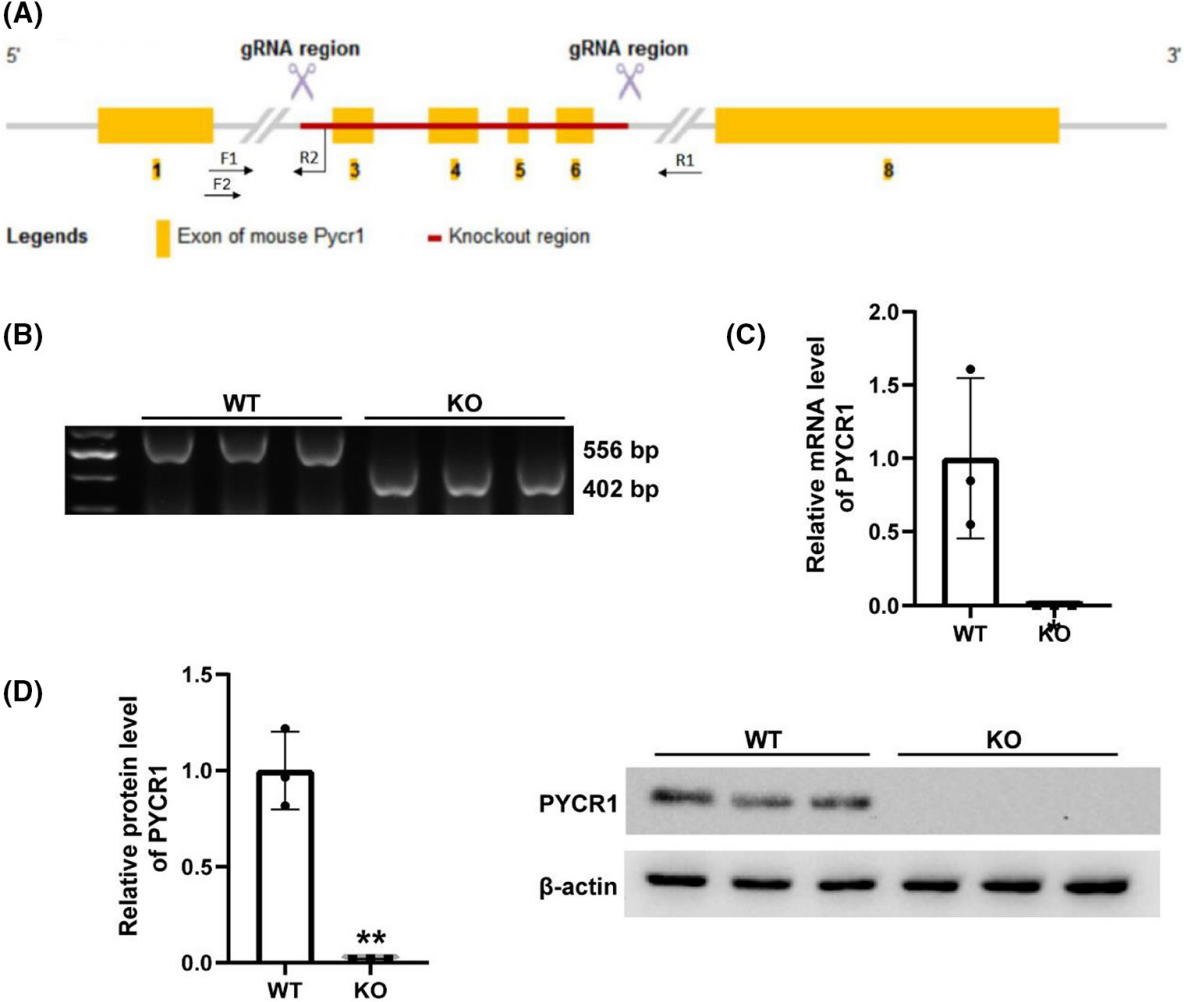
CRISPR/CAS9‐mediated KO of Pycr1 in mice. (A) Genotyping strategy for producing Pycr1 KO mice. (B) PCR screening and analysis for genotyping Pycr1 KO mice. (C) qRT–PCR for Pycr1 KO mice and WT control. (D) Western blot analysis and quantification of Pycr1 expression in the WT and KO groups. The data are presented as the mean ± SD values. *p < 0.05, **p < 0.01, ***p < 0.001 versus the control group.

**FIGURE 3:**
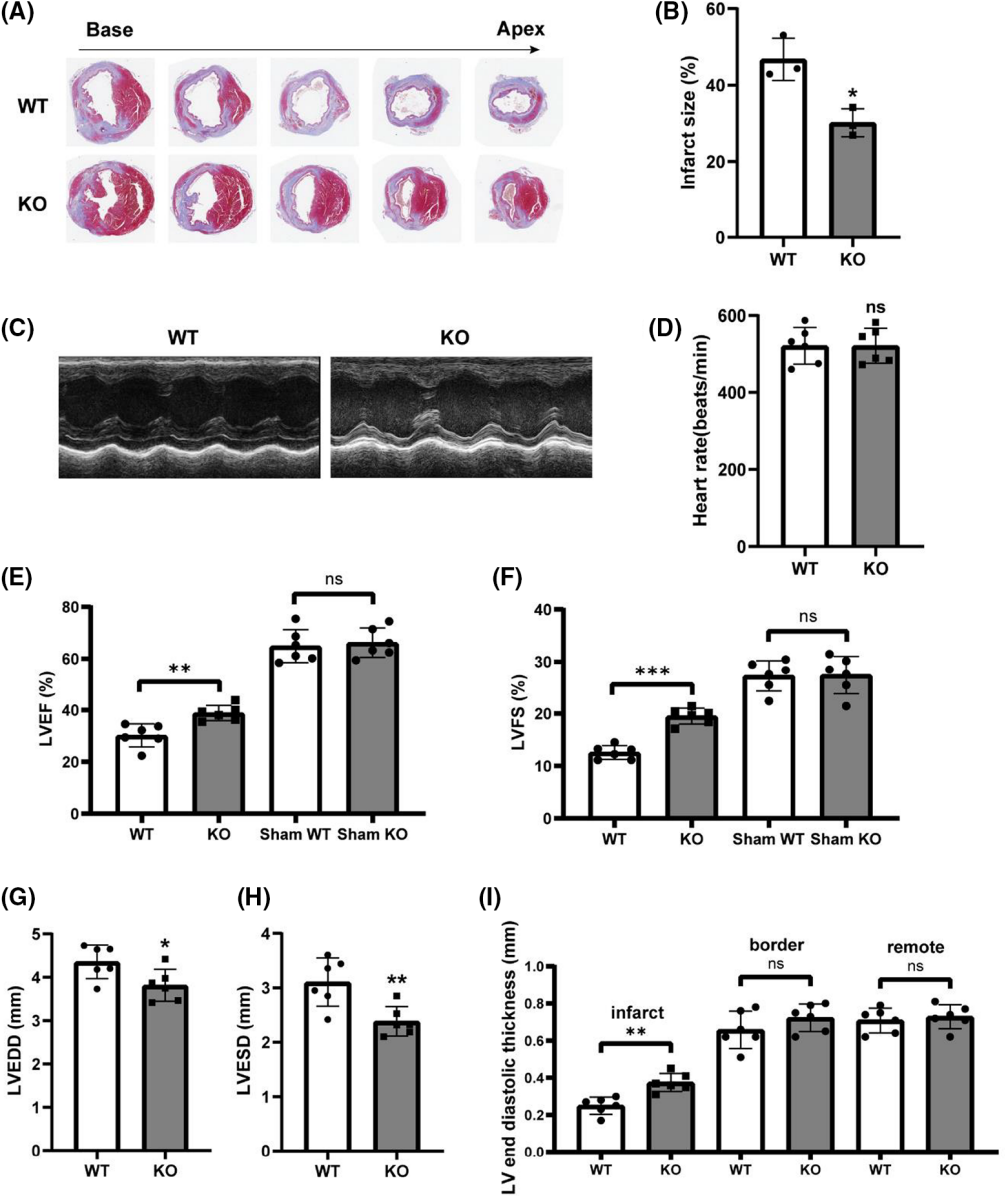
Deletion of Pycr1 via CRISPR/Cas9 improved cardiac function after MI in mice. (A,B) Representative images and quantitative analysis of myocardial slices subjected to Masson trichrome staining from the WT and KO groups. Blue staining indicates the infarct area. (*n* = 3). (C) Echocardiography indicated that Pycr1 deletion improved the cardiac function of the mice. (D) Heart rate of mice in the WT and KO groups after MI (*n* = 6). (E–I) Echocardiographic results of the LVEF, LVFS, LVESD, LVEDD, and LV end diastolic thickness for mice in the WT and KO groups after MI or Sham (*n* = 6). *p < 0.05, **p < 0.01, ***p < 0.001 versus the control group.

We characterized the KO efficiency of the Pycr1 gene at three levels: DNA, RNA and protein. Figure 2A shows the strategy of constructing Pycr1 KO mice with CRISPR/Cas9. Figure 2B shows the expression of mutant fragments in KO mice, and panels c and d show that the mRNA and protein expression levels in KO mice were significantly decreased. To determine whether Pycr1 KO after MI has a protective effect, Masson's trichrome staining to mark the infarct zones, which were stained blue, showed that the infarct size and myocardial fibrosis were reduced in the KO group compared to the WT group (Figure 3A,B). After ligation of the LAD, we used echocardiography to evaluate left ventricular function. The typical M‐shaped echocardiographic image is shown in Figure 3C—LVEF, LVFS were significantly higher in KO mice than in the MI mice, while LVEDD and LVESD in KO mice were significantly lower than those in the WT mice (Figure 3E–H). However, the heart rate remained unchanged between the two groups (Figure 3D). For LV end‐diastolic thickness, the KO group mice were higher than WT mice in the infarct region, and there was no difference between the border and remote regions. (Figure 3I).

### 3.3 Metabolomics analysis of the cardioprotective effect of Pycr1 KO against MI

**FIGURE 4:**
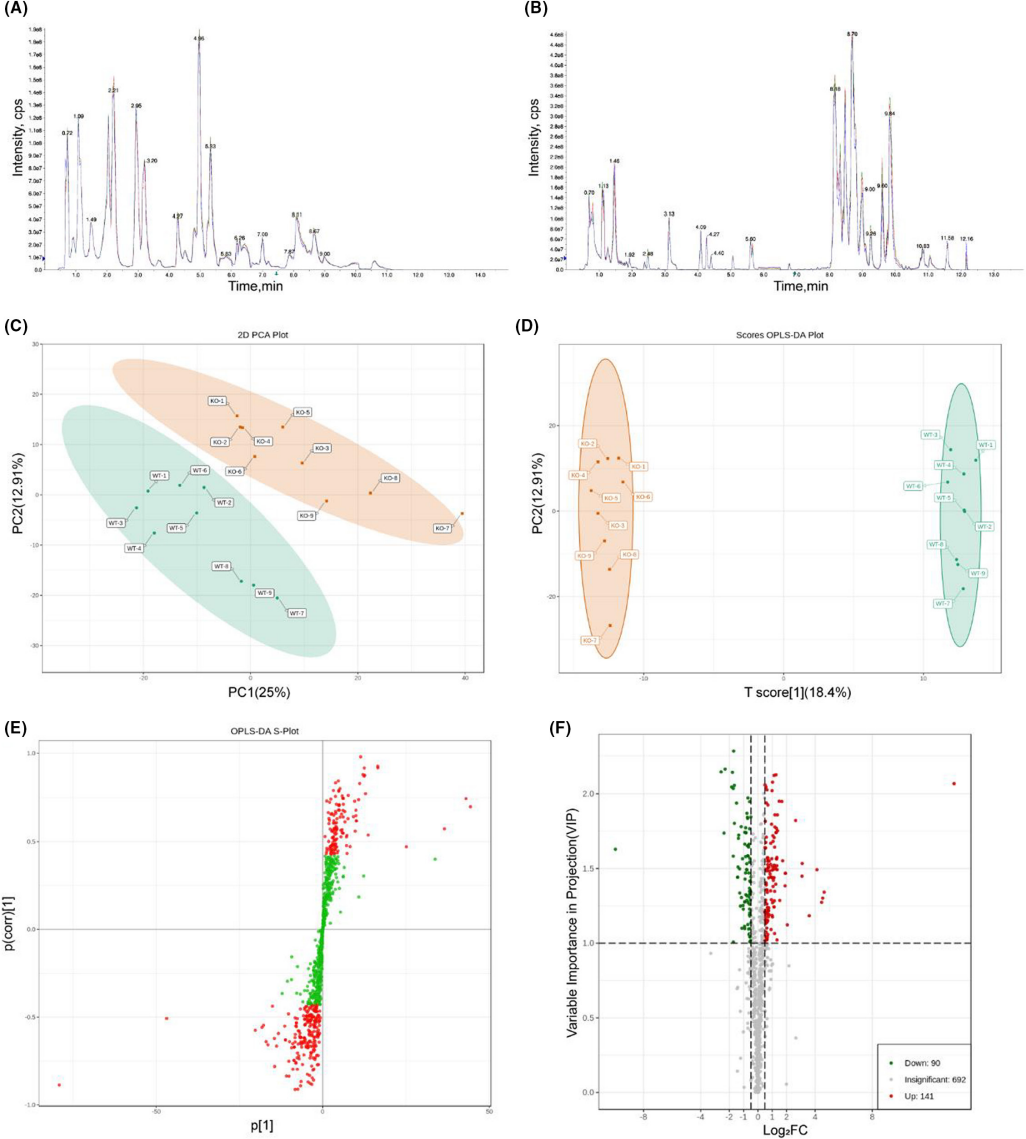
Metabolomics analysis of the hearts of mice in the WT and KO groups. (A,B) The total ion current (TIC) of the mixed QC sample used for quality control. (C) PCA score plot: the WT group is green, and the KO group is red. (D) Score plot of the OPLS–DA model between the WT and KO groups. (E) S‐plot of the OPLS–DA model between the WT and KO groups. (F) Volcano plot of the differential metabolites between the WT and KO groups.

Analytic 1.6.3 software was used to process the mass spectrometry data. The figure shows the total ion flow plot of the mixed QC sample (total ion current, TIC, that is, the intensity of all ions in the mass spectrogram at each time point is added and continuously depicted), the abscissa is the retention time (retention time, Rt) of the metabolite detection, and the ordinate is the ion flow intensity of the ion detection (intensity unit is cps, per count second) (Figure 4A,B). PCA is the method most commonly used to reduce the dimensionality of multivariate datasets through data decomposition. The PCA score map showed that the sample points obviously tended to gather in their groups (Figure 4C). OPLS–DA was used to optimize the overall discriminant model, and the OPLS–DA score map (Figure 4D) shows the complete separation between the WT and KO groups. The s‐diagram of the OPLS–DA model was used to screen differential metabolites. The horizontal axis represents the correlation coefficient between principal components and metabolites, and the vertical axis represents the correlation coefficient between principal components and metabolites. Metabolites that are plotted closer to the lower right and lower left corners exhibit more significant differences between groups. Red dots indicate that the VIP value of the metabolite is greater than or equal to 1, and green dots indicate that the VIP value of the metabolite is less than 1 (Figure 4E). For the screening of differential metabolites, metabolites with log2‐fold change values greater than 0.5 and VIP values greater than 1 met the screening standard, and a total of 231 differential metabolites were obtained, of which 141 metabolites increased and 90 metabolites decreased. The difference in metabolite expression levels between the two groups and the statistical significance of the difference were visualized in a volcano plot for easy observation (Figure 4F).

**FIGURE 5:**
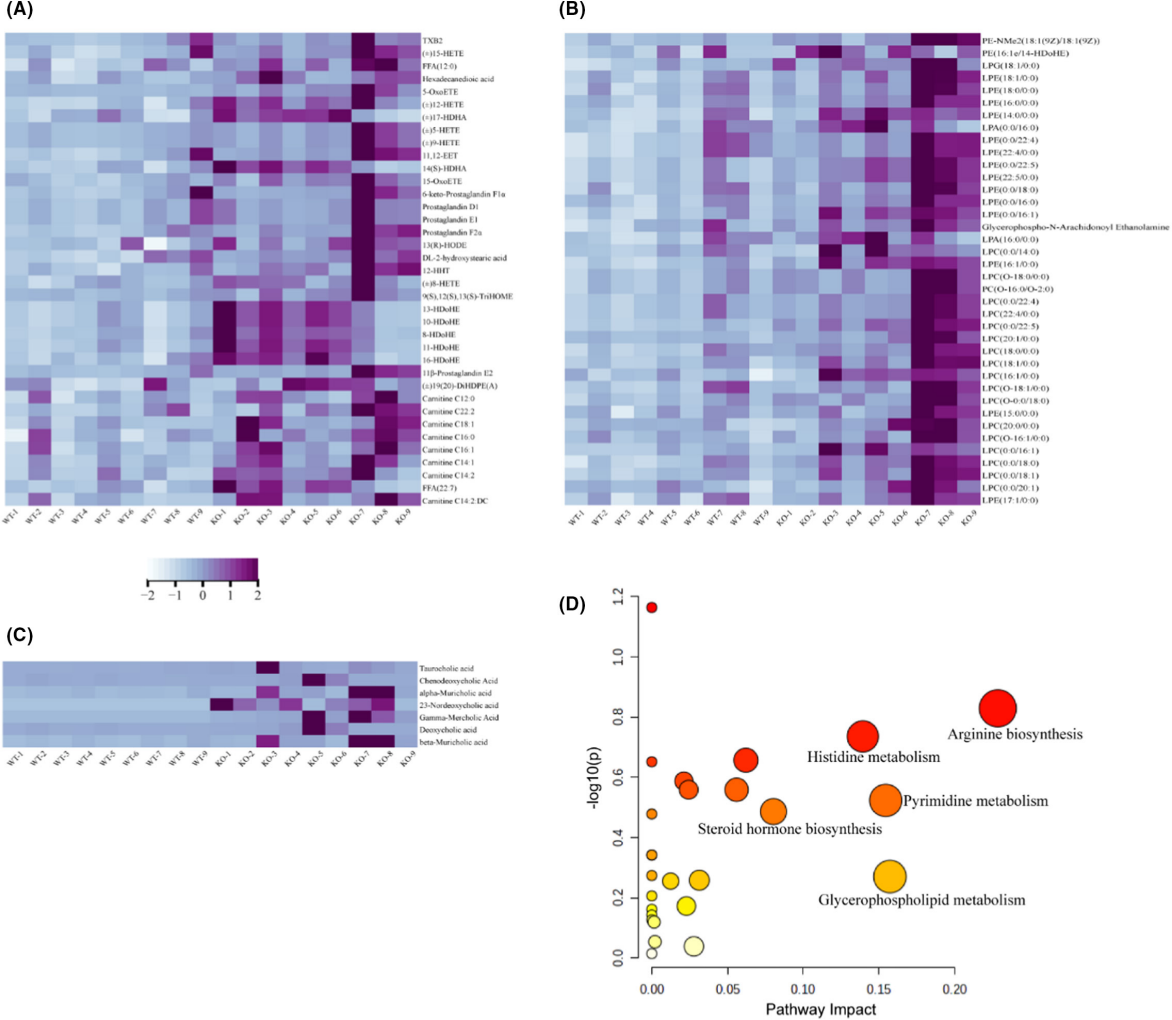
Metabolomics analysis of the cardioprotective effect of Pycr1 KO after MI. (A–C) Heatmap showing the differentially expressed primary metabolites in fatty acid, glycerol phospholipid and bile acid metabolism. (D) Metabolic pathways identified by MetaboAnalyst for primary metabolites differentially between WT and KO mice.

After the classification and analysis of the differential metabolites from the above screening, we found that among the primary metabolites, the contents of fatty acids, glycerol phospholipids and bile acids increased significantly in the hearts of the Pycr1 KO mice with MI. Through cluster heatmap analysis, we identified 37 fatty acid metabolites with elevated content, including TXB2 (Figure 5A); 38 glycerol phospholipid metabolites with increased content, such as PE‐NMe2 (Figure 5B), and 7 bile acid metabolites, such as taurocholic acid, with increased content (Figure 5C). Metabolic pathway enrichment analysis of the identified differential metabolites was performed with the MetaboAnalyst (https://www.metaboanalyst.ca) tool, and the metabolic pathways altered after MI in KO mice compared with the WT group included arginine biosynthesis, histidine metabolism, pyrimidine metabolism, glycerophospholipid metabolism, and steroid hormone biosynthesis, among others (Figure 5D).

### 3.4 Transcriptomics analysis of the cardioprotective effect of Pycr1 KO after MI

**FIGURE 6:**
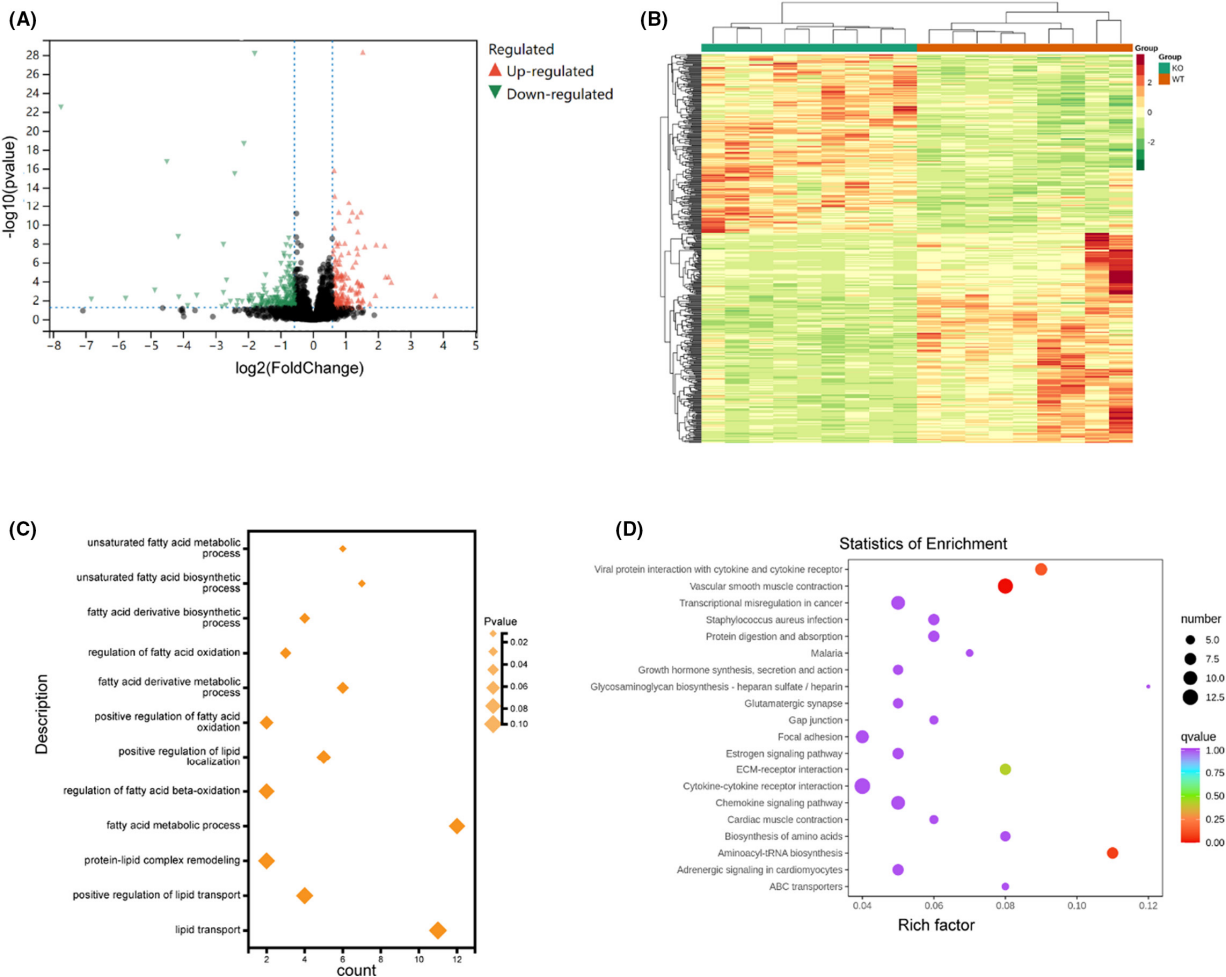
Transcriptomics analysis of the cardioprotective effect of Pycr1 KO after MI. (A) Volcano map, *X*‐axis: logarithm of the fold‐change between two groups, *Y*‐axis: −log10 of FDR of the difference between two groups. (B) Heatmap. Each column in the figure represents a sample, and each row represents a gene. The gene expression in different samples is represented by different colours, with red representing high expression and blue representing low expression. (C) GO enrichment bubble diagram of differential genes. (D) KEGG annotation diagram of differential genes.

RNA expression levels are an important reflection of gene expression regulation. Therefore, we used transcriptomics methods to study the changes in the expression of heart mRNA in Pycr1 KO mice after MI. According to the criteria of | log_2_Fold change| > 0.5 and FDR < 0.05 to define differential gene expression, we screened out 215 upregulated genes and 247 downregulated genes, which were displayed in a volcano map to visualize the overall distribution of differential genes in the two groups of samples (Figure 6A). The centralized and standardized FPKM expression of differential genes was extracted, hierarchical cluster analysis was performed, and the cluster heatmap of each difference group was plotted (Figure 6B). GO is an international standard classification system for gene function. The database established by the GO Consortium aims to establish a linguistic vocabulary standard that is applicable to various species, defines and describes the function of genes and proteins, and can be updated as research progresses. After identifying the differentially expressed genes, enrichment analysis was performed to study the distribution of these genes in GO categories to characterize the differences in the gene function between samples, and GO term significance enrichment analysis was performed based on the GO terms in the GO database. The hypergeometric test was applied to find the GO terms that were significantly enriched in the differentially expressed genes compared with the whole genome background, which included unsaturated fatty acid metabolic process (Figure 6C). Scatter plots were constructed as a graphical representation of the KEGG enrichment analysis results. In this figure, the degree of KEGG enrichment was measured by the Rich factor, *q*‐value, and the number of genes enriched in this pathway. where the Rich factor refers to the ratio of the number of genes in the pathway identified as differentially regulated in the sample to the total number of annotated genes. The larger the Rich factor is, the greater the degree of enrichment. The smaller the *q*‐value is, the more significant the enrichment. We selected the 20 most significant path entries to display in the graph; if there were fewer than 20 enriched pathway entries, all of them were displayed (Figure 6D).

## 4 DISCUSSION

The above study shows that Pycr1 mRNA and protein expression levels are reduced in the hearts of mice after MI. We constructed Pycr1 KO mice by CRISPR/Cas9 technology to demonstrate that Pycr1 gene KO has a protective effect against MI, reducing the area of the MI and improving heart function after MI. Using state‐of‐the‐art transcriptomics approaches, we found 215 upregulated genes and 247 downregulated genes after KO of the Pycr1 gene, which can affect unsaturated fatty acid metabolism at the transcriptional level. Metabolomics results revealed 141 metabolites with elevated content and 90 metabolites with decreased content, among which fatty acids, glycerol phospholipids, bile acids, and other metabolites increased significantly. The changes in the levels of these metabolites may be related to the protective effect of Pycr1 KO on the heart after MI.

It is well known that lipid metabolism in the heart and throughout the body is closely related to the occurrence and development of coronary heart disease.^12^, ^13^, ^14^, ^15^ Studies have shown that the heart is a highly energy‐intensive organ that needs an adequate supply of blood oxygen to maintain normal function. In general, 60%–90% of the energy required for myocardial activity comes from free fatty acids, and the other 10%–40% of the energy is provided by the metabolism of carbohydrates (glucose, lactic acid, and ketone bodies).^16^, ^17^ MI is a local myocardial ischemia caused by coronary artery occlusion, which causes decreased expression of fatty acid oxidase‐related genes and decreased fatty acid utilization in terms of energy metabolism.^18^, ^19^ In this study, we found that this metabolic change may be reversed in Pycr1 KO mice due to increased utilization of fatty acids and thus altered energy metabolism. We also observed that many of the oxidized lipid products also changed, and these products have been shown to play an important regulatory role in atherosclerosis and coronary heart disease.^20^, ^21^, ^22^ Previous studies have shown that inhibiting the fatty acid oxidase carnitine palmitoyltransferase‐I (CPT‐I) can reduce ventricular reconstruction and improve heart function,^23^ and we have also found that the recovery of cardiac function after MI in Pycr1‐knockout mice is associated with a decrease in the fatty acid oxidation levels; these findings lay a solid foundation for future research on fatty acid metabolism after MI.

In particular, we observed an increase in phospholipid content in Pycr1 KO mice after MI, and recent studies have shown that phospholipid metabolism is quite strongly related to the occurrence and development of coronary heart disease; thus, phospholipid metabolism serves both as a novel diagnostic marker of MI and as a new therapeutic target.^24^, ^25^, ^26^, ^27^, ^28^, ^29^ Studies have shown that myocardial‐specific overexpression of the small glycerol phospholipid hemolytic phosphatidic acid (LPA) can improve myocardial function and promote heart regeneration after MI.^30^ We also observed that LPA was elevated after Pycr1 KO, which may also be one of the ways in which loss of Pycr1 exerts a protective effect. In addition, we observed elevated levels of bile acids in Pycr1 KO mice. As a metabolite, bile acids not only promote the absorption of fat‐soluble nutrients but also regulate many metabolic processes, including the homeostasis of glucose and lipids. A growing body of research suggests that bile acid signalling pathway activation reduces inflammation and improves heart function after infarction, and strategies to control bile acid metabolism and its associated signalling to improve the inflammatory response may be beneficial for patients with MI.^31^, ^32^, ^33^, ^34^, ^35^ Recent studies of UDCA and its taurine conjugate taurine deoxycholic acid (TUDCA) have demonstrated the clinical potential of these compounds as anti‐apoptotic drugs in the treatment of acute MI.^36^ The metabolism of arginine is closely related to the metabolism of proline, and arginine is partially involved in the synthesis of proline. An increasing number of studies have investigated arginine in MI. Some researchers hypothesize that improving arginine metabolism has a therapeutic effect on MI,^37^, ^38^, ^39^, ^40^ but there remains a lack of direct evidence for arginine metabolism and MI. Our study found that arginine metabolism was also altered in PYCR1‐knockout mice after a reduction of endogenous proline production, which would provide indirect evidence for subsequent studies on the role of arginine metabolism in MI.

The limitation of our study is that we have not yet determined the relationship between the reduction of endogenous proline metabolism after Pycr1 knockout and the overall cardiac metabolism, which will lay the foundation for the subsequent understanding of the mechanism through which Pycr1 knockout improves the recovery of MI. When investigating the effect of Pycr1 on proline metabolism, we should not limit ourselves to the effect of expressing or knocking out Pycr1 on proline metabolism but also further test the condition of heart function after Pycr1 knockdown, which will further clarify the effect of Pycr1 on heart function after MI. Using metabolomics and transcriptomics data, we should analyse whether changes in a gene or metabolite exhibit a linear relationship with cardiac function in the future, and this potential relationship will establish a solid bridge between MI and cardiac metabolism.

## 5 CONCLUSION

In this study, we analysed GEO datasets associated with MI and found that Pycr1 RNA expression in the heart decreased after MI in mice. Western blot analysis of mouse heart tissue in an MI model demonstrated that Pycr1 protein levels in the heart decreased after MI in mice. Then, we constructed Pycr1 KO mice by CRISPR/Cas9 technology to prove that Pycr1 gene KO has a protective effect on MI, reducing the area of MI and improving heart function. Using state‐of‐the‐art transcriptomic and metabolomic techniques, we found that Pycr1 gene KO affects unsaturated fatty acid metabolism at the transcriptional level, increasing the levels of metabolites such as fatty acids, glycerol phospholipids, and bile acids to play a protective role at the metabolic level in the heart after MI. In conclusion, our research will lay a solid foundation for the development of future Pycr1‐related drug targets.

## AUTHOR CONTRIBUTIONS

**Zhimin Xue:** Conceptualization (equal); investigation (equal); resources (equal); software (equal); writing – original draft (lead). **Yiwen Pan:** Data curation (equal); formal analysis (equal); methodology (equal); software (equal). **Xugang Kong:** Investigation (equal); software (equal); supervision (equal); validation (equal). **Jiefang Zhang:** Data curation (equal); validation (equal); visualization (equal); writing – original draft (equal). **Danyu Wu:** Investigation (equal); methodology (equal); project administration (equal); software (equal). **Binquan Zhou:** Conceptualization (lead); funding acquisition (equal); project administration (equal); writing – review and editing (equal).

## CONFLICT OF INTEREST

All the authors declare that they have no competing interests.

## Data Availability

The data used to support the findings of this study are available from the corresponding author upon reasonable request.
